# Temperature extremes, climate change and multimorbidity: A rapid scoping review

**DOI:** 10.1016/j.joclim.2025.100452

**Published:** 2025-07-29

**Authors:** Hajira Dambha-Miller, Uzayr Nagdi, Lucy Smith, Glenn Simpson

**Affiliations:** Primary Care Research Centre, University of Southampton, Southampton, United Kingdom

**Keywords:** Temperature extremes, Climate change, Multimorbidity, Health outcomes, Rapid scoping review

## Abstract

**Introduction:**

Exposure to extreme temperatures disproportionally impacts vulnerable populations, including those with multimorbidity (i.e., people living with two or more long-term health conditions). A greater frequency of temperature extremes such as heatwaves driven by climate change will likely increase adverse health outcomes in vulnerable populations. Therefore, it is important to understand the potential effects of temperature extremes on the health outcomes of multimorbidity populations to aid the planning of healthcare systems and preventive interventions. In this review, evidence was collated and summarised, describing the health outcomes of extreme temperatures amongst people with multimorbidity.

**Methods:**

A rapid scoping review with searches on temperature extremes and outcomes in multimorbidity populations was conducted using Medline, CINAHL, Scopus and Wiley Library. These searches were supplemented with manual citation and Google Scholar searches. There were 1,225 titles screened, with data extracted by two independent reviewers. Eight papers were included in the final analysis.

**Results:**

Relatively few studies were identified, indicating limited evidence on this topic. Existing evidence focused on the increased risk of mortality in the multimorbidity population from extreme heat. No studies were identified examining the impact of cold extremes on the health outcomes of those with multimorbidity.

**Conclusion:**

There is a need for significant further research, including systematic review and/or empirical investigation, on a range of issues that can further understanding of the effects of temperature extremes on health outcomes of multimorbidity populations.

## 1 Introduction

Multimorbidity refers to the co-occurrence of two or more chronic long-term health conditions in an individual [1]. It impacts over half of adults worldwide aged over 60 years old and is increasing in prevalence [2]. Multimorbidity presents a complex challenge to health systems globally reflecting an intersection of population longevity and the rising prevalence of long-term health conditions [2]. People with multimorbidity usually experience a range of health complications and wider care challenges as a result of multiple conditions that often ‘adversely interact with each other’ which heightens the vulnerability of this cohort to a range of external environmental stressors [1].

There is no universally accepted definition of a temperature extreme event, with different disciplines adopting varied approaches and perspectives on this phenomenon. Temperature extremes, whether heat or cold-related, are usually characterised by significant deviations from typical seasonal temperature thresholds experienced in a particular geographical location or climate zone [3]. Typically, temperature extreme events manifest as intense periods of heat (i.e., heatwaves) or severe cold events that occur over a period of time (e.g., three days or more) [3]. Climatological measures of temperature extremes are commonly employed, based on percentiles, which can be used to assess associated health risks [4]. For example, definitions of extended periods of extreme heat have been characterised as exceeding the 90th percentile of temperature thresholds, whilst cold events can be defined by sustained temperatures below the 10th percentile [4]. In relation to health impacts, ‘a heat (cold) wave can be considered as a period with sustained temperature anomalies resulting in one of a number of health outcomes, including mortality, morbidity and emergency service call-out’ [5]. Furthermore, factors such as ‘wave intensity and duration, but also time of the year, are important determinants of the impact on health’ [5].

Previous research indicates a shifting landscape in climate patterns resulting from anthropogenic-driven global warming that is increasing in frequency, duration and intensity of temperature extremes globally [3]. Not only do these extreme temperature events disrupt daily life patterns, but more importantly, they pose significant health risks to individuals, especially among those with a pre-existing long-term chronic health condition who are more vulnerable to the effects of temperature extremes [6]. The evidence shows that the impacts of temperature extremes on human health are multifaceted and result from a range of ‘complicated mechanisms’, which are often difficult to identify and measure accurately to determine cause and effect [6]. It is important to note that whilst heatwaves garner significant attention, especially in the context of global warming, cold episodes continue to present equally significant health challenges [7], in particular, by exacerbating pre-existing long-term respiratory, cardiovascular, and cerebrovascular conditions that are common in multimorbidity [[7], [8], [9], [10]]. Therefore, multimorbidity populations, who are living with and managing co-occurring multiple long-term chronic conditions, are at increased risk of poor health outcomes. For example, this could increase medical presentations and hospitalisations, which in turn could further exacerbate long-standing seasonal challenges on healthcare systems such as winter pressures, and increasingly ‘summer pressures', in temperate zones, as a result of heatwaves induced by human-driven climate change [[11], [12], [13]].

Despite the growing body of literature examining the harmful health impacts of temperature extreme events, there remains a significant gap in evidence that comprehensively describes these effects on multimorbidity populations. This is significant in the context of the predicted growth of multimorbidity across all population groups and countries globally [1]. The recent unprecedented shifts in climate patterns demand a greater understanding of the nexus between multimorbidity and temperature extremes, to guide and support planning in healthcare systems, facilitate the development of preventive healthcare strategies and inform the design of tailored care interventions for this cohort [13,14]. This review aimed to collate, summarise and interpret existing evidence relating to the impact of temperature extreme events on the health outcomes of people with multimorbidity.

## 2 Methods

### 2.1 Rapid scoping review approach and focus

A rapid scoping review was conducted to gather a range of heterogeneous literature on the impacts of extreme temperatures on people with multimorbidity [15]. There is no standardised definition of a rapid scoping review. However, such reviews are a form of evidence synthesis that use more streamlined methods compared to the standard systematic review methodology to enable the generation of knowledge within a limited timeframe [16]. This can be in response to immediate policy or research priorities or unexpected events, where time is short and robust evidence is needed quickly, such as occurred during the global Covid-19 pandemic [17]. The scoping review method allows for the inclusion of diverse study methodologies and quality, which can still be systematically synthesized and reproduced, whilst also identifying gaps in the existing evidence base.

This review follows established guidelines for conducting scoping reviews and is reported using the PRISMA-ScR checklist [18].

### 2.2 Search strategy

Preliminary searches were conducted on BASE (Bielefeld Academic Search Engine) and Web of Science to help develop the search strategy and gain familiarity with the literature in this field. Four electronic databases were searched including Medline (EBSCO), CINAHL, Wiley Library and SCOPUS, from database inception to January 2024. This was supplemented with manual citation and grey literature searches using Google Scholar and searches of references in extracted articles. Search terms including Medical Subject Heading (MeSH) terms, free text, and appropriate synonyms were used across the various databases (Table 1 in Supplementary Materials).

### 2.3 Eligibility criteria

Studies were eligible for inclusion if they were written in the English language, included adults over 18 years living with multimorbidity (i.e., the presence of two or more long-term chronic health conditions), and contained relevant evidence of the impact of temperature extremes on the health of multimorbidity populations [1,19]. No restrictions were applied to study design or quality.

### 2.4 Study selection and data extraction

Two reviewers independently screened all titles, abstracts, and then the full texts using the Rayyan online collaborative review screening platform [20]. Uncertainty or disagreements between reviewers at any stage were resolved through discussion, and if consensus could not be reached, a third reviewer was consulted. Reasons for exclusion at the full-text screening stage were documented. Data were extracted from full-text review into a MS Excel spreadsheet including the study year, location, population, sample size, research design, primary aim of the study, key findings and limitations of the study.

The data charting technique was used to systematically collate and extract information relevant to the research focus of this study [[21], [22]]. This involved the key data from the relevant articles being extracted into a data charting table. As each paper was reviewed, key characteristics relevant to the review’s research question were systematically identified by sifting and sorting the material [22]. Once all the extracted articles were reviewed, the research team reassessed each paper to ensure all relevant data were extracted.

This information is collated and summarised in Table 2, which enables comparison between the included studies.

### 2.5 Data analysis

The data collected were analysed using standard scoping review methodologies [21]. Descriptive counts were used to summarise key characteristics, such as the number, type, and quality of studies identified. The analysis was conducted iteratively with the extracted data descriptively interpreted by the study team [[21], [22], [23]]. Table 2 (Supplementary Materials) lists the extracted data and provides a summary of each included article.

## 3 Results

### 3.1 Screening and study selection process

The searches conducted yielded a total of 1,225 studies and after removal of duplicates, 844 articles were screened for titles and abstracts. The title and abstract screening process resulted in 132 full texts being reviewed in more detail. Of these, eight final papers were included in this rapid scoping review.

**Fig. 1:**
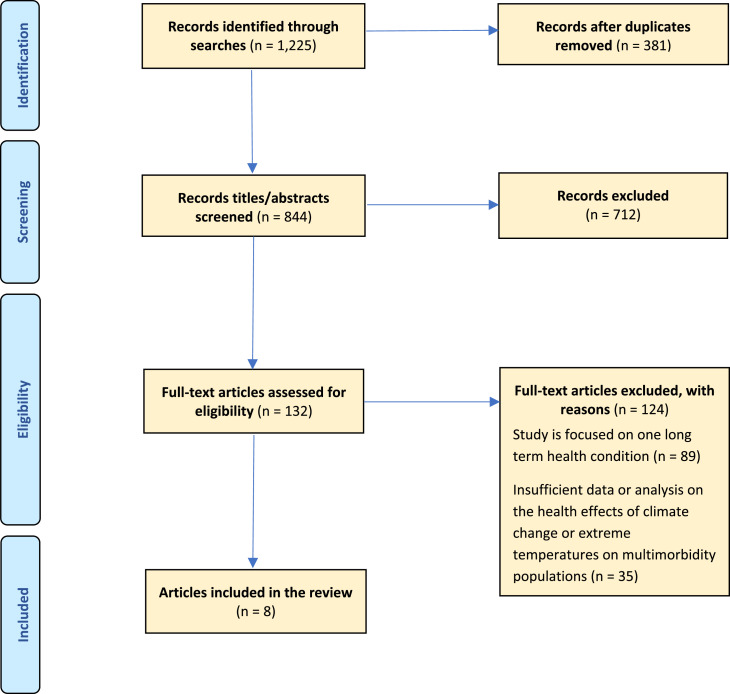
Adapted PRISMA flow chart explaining the study’s documentary inclusion process.

A PRISMA flow diagram setting out the screening and study selection is shown in Fig. 1.

### 3.2 Characteristics of included studies

A summary of the included study characteristics is shown in Table 2. These were carried out between 2007 and 2023. They included studies from Australia (*n* = 3) [[24], [25], [26]], Canada (*n* = 2) [27,28], Italy (*n* = 1) [29], China (*n* = 1) [30], and Europe as a whole (*n* = 1) [31].

All studies examined heat events on health outcomes in multimorbidity populations and attributed this mainly to climate change or global warming [24–30]. Among these, studies (*n* = 4) investigated single heatwave events and the characteristics of individuals at higher risk during these occurrences [24,25,27,29]. Four studies reported that older people living with various multiple long-term conditions were at increased risk of heat-related impacts, especially mortality, during heatwave events [24,26,29,31]. Further, studies (*n* = 4) measured heat-related mortality as an outcome [[24], [25], [26], [27], [29]]. Individual studies examined heat-related morbidity and mortality [28], hospitalisation and mortality [31], and heat-related morbidity and the number and accumulation of long-term conditions [30].

No studies were found examining extreme cold events in the context of multimorbidity.

### 3.3 Assessment of the evidence of included studies

Coates et al. [26] analysed a large dataset covering all heat-related deaths in Australia over a relatively long period, although this study had limitations, including potential inaccuracies in coronial records. Similarly, Foroni et al. [29] conducted a case-control study in Modena City, Italy, during a European heatwave, focusing on older adults aged 70 and over. While this study provided valuable insights, its findings do not fully represent the broader adult population. On the other hand, Lee et al. [27] used a large sample size and robust statistical analyses to confirm the increased mortality risk during extreme heat events, employing a meticulous methodology to minimise confounding factors. Zhang et al. [25] and Hu et al. [30] also supported the theme of increased mortality risk indirectly through case-control studies, although each had its own methodological considerations such as potential recall bias and limitations in the definition of chronic diseases. Yardley et al. [28] conducted a literature review, highlighting the vulnerability of individuals with multimorbidity during extreme heat events, but noted the variability in the quality of the studies included, which could have affected the reliability of the conclusions. Finally, Martinez et al. [31] synthesised findings from various literature reviews, emphasizing the vulnerability of individuals with specific chronic diseases during extreme heat events, particularly in Europe, although the reliance on secondary data and the regional focus may limit the generalisability of their findings.

Lee et al. [27] demonstrated that the odds of mortality during an extreme heat event in Canada were higher among individuals with more chronic diseases, particularly those with three or more long-term conditions. Similarly, Foroni et al. [29] found that individuals who died during the 2003 heatwave in Italy had a high degree of multimorbidity and were more likely to be taking multiple medications. Martinez et al. [31] also found that medication prescribed for a range of long-term conditions can increase risks related to heat exposure.

Coates et al. [26] analysed heat-related deaths in Australia and observed that most of those who died were found to have ‘multiple disabilities’, including both physical and mental long-term health conditions. While statistical analysis was not conducted, the study concluded that pre-existing health conditions were a risk factor for death during heatwaves. In contrast, Pham et al. [24] found conflicting results in their analysis of an Australian heatwave, suggesting no significant difference in comorbidity burden between ‘high-risk’ and ‘non-high-risk’ groups during the event.

Four papers highlighted the heightened risk faced by individuals with diabetes and associated long-term conditions during extreme heat events [26], [27], [28], [31]. Yardley et al. [28] discussed how diabetes can impair the body's ability to dissipate heat effectively, particularly in combination with hypertension and cardiovascular disease. This impairment in the body’s ability to regulate temperature can increase the risk of heat-related morbidity and mortality in multimorbidity populations. Martinez et al. also observed that those with multiple chronic conditions, particularly elderly people, are at greater risk during heatwave events due to ‘dysfunctional thermoregulatory mechanisms’ [31].

Zhang et al. [25] found that several coexisting long-term conditions typical in multimorbidity populations, including kidney disease, heart disease, dementia, and depression, could be related to an increased risk of having heat-related hospitalisation.

Hu et al. [30] conducted a longitudinal study examining the relationship between climate change, air pollution, and longitudinal changes in multimorbidity among those aged over 45 years. This study found that increasing temperature in association with exposure to particulate matter (PM2.5) exacerbated multimorbidity over time. Significantly, this study also showed that temperature was more harmful to multimorbidity than exposure to PM2.5, with rural residents having a higher prevalence of multimorbidity related to temperature. This may indicate that rural lifestyles and air pollution contributed to greater exposure to heat health effects during extreme events.

Martinez et al. [31] found evidence that there was a need to develop interventions applicable to indoor settings in response to heatwave events. These interventions included ‘passive cooling’ approaches, such as housing adaptations, and ‘active cooling’ technologies like air-conditioning and personal cooling devices. This study also highlighted the limited understanding of the ‘thermal comfort needs’ of vulnerable populations, including multimorbidity populations [31]. Similarly, Zhang et al. [25] identified several factors and interventions that could reduce risks during heatwaves, such as engaging in more social activities, attaining a higher level of education, having an air conditioner in the bedroom, utilising emergency buttons, and having access to refreshments.

However, Martinez et al. [31] highlighted the need for awareness of inherent social inequalities such as inequitable access to adaptive measures and technologies. For example, air conditioning can be unaffordable to many people and organisations, being costly to install and operate. Further environmental and social impacts can also occur through increasing demand on energy generation networks during periods of extreme heat, potentially increasing strain on these systems.

Whilst the evidence gathered suggests that extreme heat was associated with or contributed to poorer health outcomes, some contradictory evidence was found. For example, Pham et al. [24] found that the burden of chronic diseases did not significantly impact mortality during a specific heatwave in Australia. However, this study had limitations such as potential bias due to exposure misclassification and a limited sample size.

This rapid scoping review did not find studies using qualitative techniques or any policy or practice evidence. Overall, variations in study design, methodologies, and population samples make comparison of findings difficult, factors that need to be considered when making wider generalisations from the results of this study.

## 4 Discussion

A rapid scoping review was conducted using a comprehensive search strategy across multiple databases supplemented by manual and generic searches for completeness. To the authors’ knowledge, this is the first study to identify and summarise evidence on the impact of extremes of temperature on the health outcomes of people living with multimorbidity.

Overall, the evidence gathered by this rapid scoping review suggests a link between multimorbidity and an increased mortality risk among this population, specifically during heatwave events. Multimorbidity was primarily identified as a risk factor in relation to the effects of extreme heat often in combination with a range of other risk factors such as humidity, exposure to air pollution, living with cognitive disorders, and high usage of medications. Studies suggest that older people in particular are potentially at heightened risk of heat-related impacts during heatwave events. Additionally, studies indicated that particular long-term conditions may increase the risk of heat-related impacts during heatwave events among multimorbidity populations. These included long-term conditions affecting the heart (e.g., cardiovascular, cardiac and ischaemic heart disease), psychiatric conditions (e.g., psychoses, depression and schizophrenia), neurological disorders (e.g., Alzheimer’s disease and dementia) and diabetes (especially in combination with hypertension). However, the validity of these findings must be considered in the context of the limited evidence base of this rapid scoping review.

Several gaps in the current evidence base were identified, which require further research. There are important gaps in the understanding of the health impacts of temperature extremes on multimorbidity populations, especially the broader health outcomes and service impacts beyond increased mortality risk, such as levels of hospitalisation, number of outpatient appointments, emergency service utilisation, long-term use of social care and primary care services, and additional financial costs to care services. Further, there is a need for longitudinal studies exploring the long-term impacts of temperature extremes on the multimorbidity population. There is also a notable absence of studies investigating the potential health impacts of extreme cold events on the multimorbidity population which is especially relevant for countries in colder latitudes.

Future research efforts should focus on the implications of extreme temperatures for future clinical practice and care management of multimorbidity, whether prescribed medications increase susceptibility to the effects of temperature extremes, and identification of possible preventive or mitigating measures tailored to the specific care needs of the multimorbidity cohort. For example, improved advice and public health messaging targeted at those with two or more health conditions and their informal carers, and enhanced health and social care training to improve practitioner awareness of risks and possible responses [31] and the potential of adaptive technologies could be helpful. There is also a lack of qualitative studies in the current evidence base exploring issues such as the lived experiences of people with multimorbidity and their unpaid carers concerning existing and potential future impacts of extreme temperatures on health and wellbeing. Related to this, further work is needed to understand how the ‘thermal comfort’ and care needs of the multimorbidity population in the context of temperature extremes can be better managed going forward [31].

Furthermore, the increasing availability of large primary care datasets allows researchers to conduct large-scale big data analytics using millions of patient records, which has significant potential to advance understanding of the care needs of people with multimorbidity during extreme temperature events [19]. In turn, this could contribute to the design of effective interventions and mitigation measures for this cohort [32]. More research and data are needed to increase understanding of the causal mechanisms underlying the relationship between extreme temperatures and health impacts on multimorbidity populations, including which combinations of long-term conditions render individuals more susceptible to temperature extreme events.

This rapid scoping review has limitations. An important limitation was the scarcity of relevant studies identified, which limits the generalisability of the review’s findings. Whilst numerous studies were found during the screening process that explored the effects of extreme temperatures on individual long-term conditions, there was a paucity of evidence specifically related to the multimorbidity population. Another limitation is the relatively modest sample sizes of the studies included in this work. Additionally, the review encompassed various study designs and methodologies, which may affect comparability between the included papers. Significantly, no studies were identified that used qualitative techniques, which prevents a deeper understanding of phenomena such as the lived experience of the multimorbidity population during extreme temperature events. The searches conducted by this review did not find any policy or practice evidence. Further, the inclusion criterion of using studies only written in the English language may have excluded important work produced in other languages. It is plausible that ‘narrower’ search terms, or those specific to cold temperature extremes, may have yielded further evidence for inclusion in this study.

## 5 Conclusion

Using a rapid scoping review method, this study highlighted the paucity of evidence on the impact of temperature extremes on the health and wellbeing of multimorbidity populations. This work tentatively suggests that particular long-term conditions, especially in combination, including heart conditions, neurological diseases, psychiatric disorders and diabetes, may lead to a heightened risk of an individual with multimorbidity experiencing heat-related impacts during intense periods of high temperature and humidity. This study found no evidence of the effects of cold temperature extremes on multimorbidity populations. Beyond this, the limited evidence found by this review makes it difficult to draw definitive conclusions or offer broader generalisations on how extreme temperatures affect those with multimorbidity.

Therefore, this review highlights the need for significant further work on this topic such as an in-depth systematic review to identify and collate all available international research evidence, including policy evidence and grey literature sources. Further, there is a need for empirical research to address the range of substantial gaps identified in the existing evidence base, especially in light of the predicted intensification of climate change over the next decade and the potential for harmful health impacts on multimorbidity populations.

## Ethics approval and consent to participate

Ethical approval was not required for this rapid scoping review and therefore not applicable.

## Funding

HDM has received funding from the National Institute for Health and Care Research - the Artificial Intelligence for Multiple Long-Term Conditions, or "AIM". 'The development and validation of population clusters for integrating health and social care: A mixed-methods study on multiple long-term conditions' (NIHR202637); receives funding from the National Institute for Health and Care Research ‘Multiple Long-Term Conditions (MLTC) Cross NIHR Collaboration (CNC)’ (NIHR207000); and receives funding from the National Institute for Health and Care Research ‘Developing and optimising an intervention prototype for addressing health and social care need in multimorbidity’ (NIHR206431). The views expressed in this publication are those of the author(s) and not necessarily those of the NHS, the National Institute for Health Research or the Department of Health and Social Care.

## Declaration

The lead author affirms that the manuscript is an honest, accurate, and transparent account of the study being reported; that no important aspects of the study have been omitted. The opinions, results, and conclusions reported in this article are those of the authors and are independent from the funding sources.

During the preparation of this work the author(s) used Grammarly in order to improve the readability of the manuscript and to check for grammar and typographical errors. After using this tool/service, the author(s) reviewed and edited the content as needed and take(s) full responsibility for the content of the publication.

## CRediT authorship contribution statement

**Hajira Dambha-Miller:** Writing – review & editing, Writing – original draft, Conceptualization. **Uzayr Nagdi:** Writing – review & editing, Writing – original draft, Methodology, Formal analysis. **Lucy Smith:** Writing – review & editing, Resources. **Glenn Simpson:** Writing – review & editing, Writing – original draft, Methodology, Formal analysis.

## Declaration of competing interest

The authors declare that they have no known competing financial interests or personal relationships that could have appeared to influence the work reported in this paper.

## Appendix Supplementary materials

Image, application 1
