# Supplementary material for: Temperature extremes, climate change and multimorbidity: A rapid scoping review
Source: J Clim Chang Health. 2025 Jul 29;24:100452. doi: 10.1016/j.joclim.2025.100452 (PMC12851257; doi:10.1016/j.joclim.2025.100452)
Supplement: Supplementary file 1 [file mmc1.docx]

**Supplementary Materials**

**Table 1: Summary of search terms**

| **MeSH and Free Text Search Terms** | **Databases** | **Filters/Refined by** | **Number of Sources identified** |
| --- | --- | --- | --- |
| **1) MeSH**: (MH "Chronic Disease") OR (MH "Multiple Chronic Conditions")  **FREE**: multimorbidit* OR "long-term condition*" OR "chronic disease*" OR "chronic illness*"  **2) MESH**: (MH "Global Warming") OR (MH "Sea Level Rise") OR (MH "Climate Change")  **FREE**: temperature* N3 extreme* OR climate N3 change* | Medline EBSCO | MEDLINE database.  Language: restricted to the English language. | 317 + 50=  **367** |
| **1) MeSH**: (MH "Chronic Disease") OR (MH "Noncommunicable Diseases")  **FREE**: multimorbidit* OR "long-term condition*" OR "chronic disease*" OR "chronic illness*"  **2) MESH**: (MH "Global Warming") OR (MH "Sea Level Rise") OR (MH "Climate Change")  **FREE**: temperature* N3 extreme* OR climate N3 change* | CINAHL | CINAHL Plus with Full Text database  Language: restricted to the English language. | 131 + 68=  **199** |
| **1) FREE**: "multimorbidit* OR "long-term conditions" OR "chronic diseases" OR "chronic illnesses""  **2) FREE**: ""temperature extreme*" OR "extreme temperature" OR "extreme temperatures" OR "climate change" OR "climate changes"" | WILEY LIBRARY | WILEY Online Library.  Language: restricted to the English language. | **235** |
| **1) FREE**: multimorbidit* OR "multiple long-term conditions" OR "multiple chronic diseases" OR "multiple chronic illnesses"  **2) FREE**: ("climate change" OR "extremes of temperature" OR "temperature extremes" OR "extreme temperature") | SCOPUS | Language: restricted to the English language. | **263** |
|  | Searches on Google Scholar and manual searching of references | Language: restricted to the English language. | **161** |

**Table 2: Key characteristics of included studies***

| **First Author** | **Year** | **Title of Paper** | **Location** | **Study design** | **Sample size** | **Key findings** | **Limitations** |
| --- | --- | --- | --- | --- | --- | --- | --- |
| Lee, M | 2023 | ‘Chronic diseases associated with mortality in British Columbia, Canada, during the 2021 western North America extreme heat event (EHE).’ | British Columbia, Canada. | ‘Retrospective Cohort Study- comparing adults who died during the 2021 EHE in Canada (25 June - 02 July 2021) with adults who died during typical weather patterns in previous years (25 June - 02 July from 2012 -2020).’ | ‘1614 EHE deaths vs 6524 typical weather deaths, after omitting children, missing location and missing demographic information.’ | ‘The EHE group had higher proportions of decedents with schizophrenia, chronic kidney disease, diabetes, and depression than the typical weather group, as well as a higher total number of chronic diseases.  The EHE group also had lower proportions of decedents with angina, dementia, and osteoporosis.  The odds of EHE mortality were higher among those with more chronic diseases.  The ORs were higher than 1.0 for those with three or more chronic diseases, and most were statistically significant.  However, there was no clear trend of increasing ORs with an increasing burden of chronic disease; most estimates for three or more chronic diseases were similar. The ORs were null for those with 1 and 2 chronic diseases.  The ORs were increased for those with four or more chronic diseases.  Having three or more chronic diseases was also associated with increased odds of EHE death, though the odds did not increase with an increasing number of diseases.’ | ‘First, this is a case‐only study comparing individuals who died during the EHE with those who died before the EHE. The reported effect estimates cannot be interpreted as risk and protective factors as could be done for a case‐control study comparing EHE deaths with EHE survivors.  Second, typical weather deaths were drawn from the 2012 -2020 period and may not reflect the deaths that would have occurred in 2021 in the absence of the EHE.  Changes in population demographics and therapeutics may lead to different point prevalence of the chronic diseases that we examined over time.  Finally, we were not able to separate the effects of air pollution from those of heat, since BC had long periods of poor air quality in 2017.’ |
| **First Author** | **Year** | **Title of Paper** | **Location** | **Study design** | **Sample size** | **Key findings** | **Limitations** |
| Foroni, M | 2007 | ‘A Retrospective Study on Heat-Related Mortality in an Elderly Population During the 2003 Heat Wave in Modena, Italy: The Argento Project.’ | Modena City, Italy. | ‘A retrospective, case-control study of older people to observe deaths between August 1^st^ to 31^st^ 2003.  Summer 2003 witnessed an excess in heat-related mortality in the elderly population.  A sample of those older people who died during this time was compared to a sample of those older people that survived.’ | ‘394 older persons living in Modena, 197 dead (cases) and 197 survivors (controls).  Survivors were randomly selected.  Survivors were matched for age, sex and location during the same period.’ | ‘A questionnaire to collect information about demographic, social, environmental, and clinical characteristics and about causes of death was completed.  Individuals who died had a complex clinical profile: They were more likely to have cognitive problems and a high degree of comorbidity and dependence. Moreover, they took a higher number of drugs and had a greater number of hospital admissions and specialist visits in the year before death.  The ability to compensate for extreme heat and humidity becomes less efficient with aging, the presence of several chronic diseases is more likely (and their impact is exacerbated by heat); and some medications to treat chronic diseases make people more prone to health hazards from hot weather.  Both acute and chronic cardiovascular and cerebrovascular diseases were the more frequent causes of death (22.7%)  The old (75-84 years) and the very old (>85 years) were the age groups most affected (mortality).  The study also says this in the form of ‘those who were taking more drugs’ were at higher risk of mortality, again indirectly linking to those with co-morbidities being a higher risk group of heat-related mortality.’ | ‘The major limit of this study is the retrospective design; prospective studies are needed to more precisely identify risk factors of heat-related death and to test the usefulness of preventive programs.  Moreover, data were collected only during summer, so we did not have the opportunity to compare our results with other possible outcomes obtained in other periods of the year.’ |
| **First Author** | **Year** | **Title of Paper** | **Location** | **Study design** | **Sample size** | **Key findings** | **Limitations** |
| Hu, K | 2023 | ‘Rural-Urban Disparities in Multimorbidity Associated With Climate Change and Air Pollution: A Longitudinal Analysis Among Chinese Adults Aged 45+.’ | China. | ‘Quantitative longitudinal study.  This study collects four waves of the China Health and Retirement Longitudinal Study (CHARLS 2011–2018), Baseline Study: 2011.  After this baseline survey, there were three follow-up surveys in 2013, 2015, and 2018.  Data on the number of chronic conditions (up to 14), sociodemographic, and environmental factors were collected.’ | ‘21,857 respondents (52,625 observations) from 125 cities were included in this study.  Adults aged over 45 years.’ | ‘Average multimorbidity score was 1.43 at the CHARLS 2011, increasing to 2.20 by 2018. The associations between temperature and multimorbidity score are relatively stable, revealing that rising temperature is associated with a higher multimorbidity score in the same individuals (i.e., worse chronic health).  In terms of temperature, the coefficients in all models are positive. Among them, the coefficient of 3-year temperature is 0.432 (95% CI: 0.403, 0.460), larger than other models, representing that a 1°C increase in temperature rises 0.432 scores in multimorbidity.  A 1°C increase in the 3-year average temperature increased 0.394 (95% CI: 0.362, 0.426) scores in multimorbidity for rural samples, whereas the figure for urban samples is 0.106 (95% CI: 0.075, 0.137). Supplementary Figure 2 shows a similar trend of extreme heat events on multimorbidity scores, suggesting that rural residents have a higher prevalence of multimorbidity related to temperature.  More people exposed to higher PM2.5 are living with higher temperatures.’ | ‘First, the study can only identify the association between temperature, PM2.5, and multimorbidity at the city level, because they could not obtain detailed respondents’ addresses to match PM2.5 exposure and temperature data at the individual level.  Second, the relationship between air pollution, climate change, and multiple chronic diseases should be a life-course issue.  Thirdly, they used self-reported doctor-diagnosed chronic diseases as the components for multimorbidity. However, this measure may underrate the prevalence of chronic diseases among respondents with low SES because they have less access to medical diagnosis than those with high SES.’ |
| **First Author** | **Year** | **Title of Paper** | **Location** | **Study design** | **Sample size** | **Key findings** | **Limitations** |
| Pham, T | 2019 | ‘Difference in the characteristics of mortality reports during a heatwave period: retrospective analysis comparing deaths during a heatwave in January 2014 with the same period a year earlier.’ | Victoria,  Australia. | ‘A single-jurisdiction population-based retrospective cohort analysis of consecutive heat-related deaths (HRDs) reported to the CCOV between 14 and 21 January 2014 with a historical comparison group.  The number of people dying in the 2014 heatwave in Australia, compared against a control group of people the year before, not falling victim to the heatwave.  Descriptive analysis of Quantitative data.’ | ‘255 deaths were identified during the study period in 2014, of which 222 cases, where a cause of death had been established, were included in the analysis.  During the comparison period in 2013, 97 deaths were identified, and 74 cases were included in the analysis comparison period in 2013 (range: 6–14 deaths per day).’ | ‘The mean CCI score was not significantly different between HRDs and non-HRDs (t(220)=1.77, p=0.78). There were also no significant differences in the number of cases in the none, mild and moderate or severe categories between the subgroups (χ2=3.1, p=0.21). This means there is no statistically significant difference in the burden of comorbidities (as measured by the CCI) between high-risk groups and non-high-risk groups in this heatwave.  When comparing the reported deaths in 2014 and 2013 from the same time period, there was no significant difference in sex; however, age was significantly higher in 2014 (mean age: 65.0 vs 59.4 years), and this was demonstrated by the age-standardised death rates, which were higher between the 50 and 94 age groups.  Most HRDs were due to either cardiac (59%) and pulmonary (6%) diseases or a combination of the two.  The study reported similar results in terms of increased age being a risk factor and the comorbidities associated with HRDs: cardiac disease, namely, ischaemic heart disease (61.7%); psychiatric disorder (28.7%) and pulmonary disease (20.2%). These can be understood as long-term conditions risk factors.  The mean age of HRDs was 70.5 years (SD=13.8), which was significantly higher than that of non-HRDs (61.0 years: SD=22.4, t(220)=3.60, p<0.001, 95% CI 4.3 to 14.6).’ | ‘There is a well-recognised difficulty in medically identifying HRDs due to a lack of specific macroscopic and microscopic findings. This has the potential to result in differences in reporting practices where death investigations are performed by different forensic pathologists. This could lead to exposure misclassification, where inconsistencies are seen in the application of the definition of an HRD leading to the size and characteristics of HRDs being misrepresented.  The study used a historical comparison group from 2013 in order to compare the number and a limited set of characteristics of cases that had been reported to the coroner.’ |
| Yardley, J | 2013 | ‘Do Heat Events Pose a Greater Health Risk for Individuals  with Type 2 Diabetes?’ | Canada. | ‘Literature review examining existing research to address the vulnerability of individuals with type 2 diabetes (and other co-morbidities) to heat-related issues.  How type 2 diabetes and diseases associated with it (e.g., hypertension), alter the body’s normal response to heat.’ | ‘A study comparing endothelium-dependent and endothelium-independent vasodilation in individuals with type 2 diabetes (n = 29) and controls without diabetes (n = 10).  Another study found impaired responses to local heat and other stimuli in a group of type 2 diabetes patients (n = 54) compared with age- and sex-matched controls without diabetes (n = 38).’ | ‘Individuals with diabetes have higher heart disease death rates, and those with cardiac dysfunction have more difficulty increasing cardiac output to maintain elevated SkBF during prolonged heat exposure. The presence of diabetes might exacerbate impairments in SkBF found in cardiovascular disease patients, potentially leading to an increased risk of heat-related morbidity and mortality.  In summary, the combination of hypertension and diabetes may lead to greater impairments in the body's ability to regulate temperature and dissipate heat, especially during heat exposure. This can increase the risk of heat-related morbidity and mortality in individuals with both conditions​.  Long-standing, poorly controlled diabetes can cause widespread damage to peripheral and autonomic nerves, leading to a decreased ability to sense heat stimuli. This neuropathy, particularly autonomic neuropathy, can impair thermoregulatory functions such as sweating and skin blood flow (SkBF), increasing the risk of heat-related morbidity and mortality.  These co-occurring conditions with diabetes can complicate the body's ability to respond to heat stress, thus elevating the risk of heat-related health issues. The paper emphasizes the importance of considering these comorbidities when assessing the risk and managing the health of individuals with diabetes during extreme heat events.’ | ‘The studies reviewed in the paper often did not adequately match participants with type 2 diabetes with controls without diabetes of the same sex, age, body composition, and physical fitness. This inadequacy in matching could affect the validity and generalisability of the findings.  The studies examined in the review are generally limited to exploring heat loss responses in older adults with type 2 diabetes. This limits the understanding of how extreme temperature affects a broader range of individuals with diabetes.’ |
| **First Author** | **Year** | **Title of Paper** | **Location** | **Study design** | **Sample size** | **Key findings** | **Limitations** |
| Zhang, Y | 2016 | ‘Risk factors for deaths during the 2009 heat wave in Adelaide, Australia: a matched case-control study.’ | Adelaide,  Australia. | ‘Retrospective Case-Control Study.  The maximum temperatures of these 5 days were 45.7, 43.4, 43.1, 41.1 and 40.6 °C, consecutively. This 5-day period was defined as the heat wave exposure period of our study (i.e., 2009).  Cases were those who died in Adelaide over the 5-day exposure period. The reference population was those who survived the heat wave period.  Controls were sampled from the South Australian State Electoral Roll (SASER) and were matched 1:2 to cases by age (±2 years) and gender.  We used randomly generated numbers for selection of controls to ensure the controls were representative.’ | ‘We had 82 cases (including 26 hospital cases and 56 coroner cases, accounting for 37% of the total deaths) and 164 matched community controls interviewed, with a median age of 77.5 (range 26.6100.7), 72 % retired and 10 % in institutional living.’ | ‘Our findings confirm the increased risk of dying during a heat wave among those who had pre-existing heart conditions. The salient increase in the risk of deaths for patients with heart disease in our study (more than 20 times higher compared with those who did not have heart diseases).  The simple regression analysis did suggest that dementia, depression and renal disease may have significantly contributed to the risk of deaths during the heat wave.  The study found that several coexisting illnesses, including kidney disease, heart disease, dementia and depression, could be related to an increased risk of having heat-related hospitalisation.  The presence of an air-conditioner in the bedroom, more social activities, a higher education level, use of emergency buttons and refreshments reduce the risk during heatwaves.’ | ‘One of the key limitations was ‘recall bias’ from the next-of-kin, which cannot be avoided in a case-control study.  Moreover, potential biases may be generated from data collection, such as self-selection bias from the participants or interviewing bias between cases and controls.  The limitations of recruiting less than 40 % of the eligible cases were mainly due to our inability to approach next-of-kin (reasons given included too ill, very busy and personal), which should be acknowledged.’ |
| **First Author** | **Year** | **Title of Paper** | **Location** | **Study design** | **Sample size** | **Key findings** | **Limitations** |
| Martinez, S | 2021 | ‘Heat and health in the WHO European Region: updated evidence for effective prevention.’ | Europe. | ‘The report is based on the findings of several literature reviews. It presents the review’s findings in the form of an overview of relevant  recent evidence, with clear implications for the  prevention of health effects caused by heat waves.  This report also includes ‘grey literature’ in the form of technical reports and studies from government and international organisations.  This report also considers the evidence of the last decade and new insights related to the links between climate change, heat exposure and  health.’ | N/A | ‘Elderly people, people with cardiovascular disease, respiratory disease, mental health disorders and diabetes are vulnerable subgroups to heat mainly due to biological mechanisms.  People with mental health disorders had been seen to increase in ER visits during extreme heat events (EHE).  Respiratory morbidity/mortality has also been seen to increase during EHE.  Literature reviews and grey literature indicate that people with underlying health conditions such as cardiovascular and cerebrovascular disease, hypertension, COPD, kidney disease, diabetes, neurological conditions such as Alzheimer’s, and mental health conditions are vulnerable groups to heat.  As well as this, people on medication, as some medication for the diseases listed above, impairs thermoregulation  and perception of risks related to heat exposure.  Elderly people are at particular risk due to  dysfunctional thermoregulatory mechanisms (limited sweating and skin blood flow), chronic dehydration, multiple chronic diseases (especially cardiopulmonary disease, diabetes and dementia) and use of medications.    Neurodegenerative diseases like dementia and Parkinson’s disease, which are associated with old age, have also been identified as risk factors with evidence of an increase in hospitalisation during heatwaves among patients with these conditions.  Subjects with cardiovascular diseases are at greater risk during extreme heat.  Several studies have identified individuals with underlying respiratory diseases, including COPD, as being at increased risk from the adverse health effects of heat.  A study on a cohort of COPD patients found that increases in indoor and outdoor temperatures were associated with increases in daily indicators of COPD morbidity, including respiratory symptoms and rescue inhaler medication use.  Among COPD patients, symptoms can worsen in response to the hyperventilation required to disperse heat and the broncho-constrictive effects of heat.  Exposure to heat has been shown to increase the risk of hospitalisation and death among individuals with diabetes during heatwaves.’ | The study relied on secondary data based on previous systematic reviews and grey literature sources such as ‘technical reports and studies from government and international organizations.’ |
| **First Author** | **Year** | **Title of Paper** | **Location** | **Study design** | **Sample size** | **Key findings** | **Limitations** |
| Coates, L | 2022 | ‘Heatwave fatalities in Australia, 2001–2018: An analysis of coronial records.’ | Australia. | ‘Retrospective Case Series Study.  Those fatalities associated with extreme heat in Australia, as identified by a Coroner, from 2001 to 2018 were studied.  Closed case 2 records from 1 July 2000 to 30 June 2018 (i.e., financial years FY2001-FY2018) were accessed from the National Coronial Information System (NCIS) database.’ | ‘At least 473 heat-related deaths were reported to a coroner in Australia in the 18 years from July 2000 to June 2018, 354 occurred during heatwave conditions and, of these, 244 occurred within or near buildings.  Unless otherwise indicated, statistics refer to the 354 heatwave fatalities.’ | ‘In general, the most deaths per five-year age category occurred in those aged 50 years and above (Fig. 3); notably in the 85+ (n = 59; 17%) and 75-79 (n = 46; 13%) age categories. Some 243 (69%) of fatalities occurred in the 60-plus age group; of the 244 fatalities that occurred indoors, 80% (n = 196) occurred in this group. Less than ten fatalities occurred in each of the 0-4 to 40-44 age groups.  The vulnerability of older Australians to heatwaves is further shown by the fatality rate, which jumped to approximately 0.11 deaths per 100,000 population for the 50–54 and 55-59 age groups to approximately 0.19 for the 60-64 to 70-74 age groups, more sharply to approximately 0.46 for the 75-79 and 80-84 age groups and then to 0.83 for those aged 85-plus.  At least 316 (89%) of decedents had some type of disability and many had multiple disabilities: e.g., 171 (48%) had three or more disabilities. Some 302 (85% of) fatalities had one or more types of physical disability and 116 (33%) had one or more types of mental disability.  48% of the sample size had 3 or more ‘disabilities’.  The most common physical disability was a heart issue (n = 224; 63%), followed by asthma or some other respiratory issue (n = 111; 31%). Obesity, mobility, renal / kidney and diabetes issues were present in 15% to 18% of cases.  The main type of mental disability was psychiatric (e.g., psychoses, depression, schizophrenia, OCD).  Our results accord with other Australian and international studies: those more likely to die in heatwave events are the elderly, young children, people with existing medical conditions, the isolated and people who experience social and financial disadvantage.’ | ‘There is no guarantee that this method will identify all heat-related deaths that occurred FY2001-FY2018:  1. Heatwaves can exacerbate pre-existing medical conditions as well as killing outright and, often, a death is coded under the primary cause of death: e.g., cardiac arrest.  2. Coroner's or autopsy reports are required to state substantiated facts only: a heat-related death cannot be determined solely by forensic pathology. Thus, if a police report is not included, there may be no heatwave-related term to be found in the data for that case  3. A relatively large proportion of coronial cases in the more recent years of record are “open” – unavailable for viewing. The true influence of this data on the interpretations from this analysis is unknown but is likely to result in an under-estimate of the number of heatwave fatalities.  In addition, data available for analysis in accessed coronial files was limited, in that Australian jurisdictions deal with data destined for coronial files in different ways: thus, data collected varies between jurisdictions and over time. Also, even when variables of interest (e.g., the presence/absence of working air conditioning) are not mentioned in NCIS data, they may still be present. For a variable to be mentioned, it must be considered pertinent to the [narrative](https://www.sciencedirect.com/topics/social-sciences/narrative) of the police report or coronial findings. This can result in data fields containing a high proportion of cases where the variable was unknown, meaning that data presented in this report may represent a lower bound.’ |

*The text included in this table has been extracted from the relevant papers.
